# Supplementary material for: Are carbon emissions trading and green financial instruments synergistic? -Comprehensive quantitative research based on content analysis
Source: PLoS One. 2024 Mar 7;19(3):e0298601. doi: 10.1371/journal.pone.0298601 (PMC10919601; doi:10.1371/journal.pone.0298601)
Supplement: S1 Data — (ZIP) [file pone.0298601.s001.zip › Data/地级市Z_F工作报告.zip!/╝¬┴╓/░╫╔╜╩╨/└┤╫╘╣1⁄2╓┌║┼í╛┬φ┐╦╩2╛▌═°í┐_2003.docx]

各位代表：

　　我代表白山市人民政府，向大会报告工作，请予审议。

　　一、2002年工作简要回顾

　　年初以来，我们在省委、省政府和市委的正确领导下，按照四届人大三次会议确定的任务，以工业经济、城市建设和社会稳定为重点，克服困难，真抓实干，促进了经济和社会的发展。

　　国民经济运行情况。全市预计实现国内生产总值102亿元，比去年增长9％；完成财政收入6.86亿元，下降4.7％，其中市本级3.4亿元，下降1.8％；全社会固定资产投资28亿元，下降16.8％；实现社会消费品零售总额42.8亿元，增长7％。工业经济稳定增长，规模以上工业企业预计完成增加值28.7亿元，比去年增长10.2％；农业和农村经济继续保持良好发展势头，林地经济综合开发初具规模，完成农业总产值29.8亿元，增长8％；旅游业明显升温，总收入2.5亿元，增长25.5％，第三产业增加值实现31.5亿元，增长11.1％。实际利用外资403万美元，比去年增长23％。

　　基础设施建设情况。公路建设完成总投资5.7亿元，在建公路里程599公里。北线一级公路全线竣工，实现了白山高等级公路零的突破。临江老岭隧道开工建设，沈长线绕市区南线工程完成立项，松江河至天池公路完成路面铺装30公里，桦树至仙人桥、十四道沟至长白等重点项目完成年度任务。完成市中心广场、滨江西街续建、市区西部排水、仿古街改造等城市建设重点项目，金河花园、惠民小区三期工程全部竣工，喜丰花园6.3万平方米高层住宅主体封闭。“绿化美化植树年”取得实效，庭院开放，植树种草，改善了城市形象。加大了城市管理力度，对群众反映强烈、影响居民生活、有碍城市功能、有损城市形象的部位进行了综合整治，市容市貌和社区环境有了一定改观。以县城为中心，以美化环境、完善基础、增强辐射带动功能为重点的小城镇建设取得进展。全市农网改造二期工程和曲家营、双河、北江、聚宝、海岛等水电重点项目建设共完成投资2.3亿元。投资2.1亿元，建成覆盖全市的白山移动光纤通信网。

　　各项改革进展情况。坚持把改革作为加快发展的动力，铝厂、铝车轮等困难企业通过资产重组、租赁经营，强化了内部管理，生产创历史新高。嵩华纸业与中国包装纸业公司重新组建了中国包装纸业投资有限公司。轴承公司与省轴承集团合作经营达成协议。完成了靖宇娃哈哈转制经营和18户中小企业产权出售。对8户企业进行了公司制改造。通过市服装工业公司试点，积极探索了集体企业资产量化给职工、经营者持大股的有效方法。对8户劣势企业依法实施破产。市造纸厂列入国家关闭破产核呆计划，天时水泥刨花板厂破产核呆进入法律程序，预计共核呆3.95亿元。协助通化矿务局顺利完成了八道江、大湖煤矿破产工作，接收了破产矿的社会职能和离退休人员管理。农村税费改革取得阶段性成果，全市争取转移支付资金4631万元，为减轻农民负担、保证乡（镇）和村级组织正常运转、农村义务教育经费支出奠定了基础。继续深化了城镇职工基本医疗保险制度改革和医疗卫生体制改革，启动了农村卫生体制改革。以行政审批、会计核算、土地收购储备制度三项改革为突破口，强化了政府自身改革。共废止市政府规范性文件132件，取消行政审批项目290项，276项行政审批和服务项目纳入政务服务中心办理，占全部保留项目的69％。大部分行政事业单位纳入会计核算中心集中统一管理。土地收储工作开始启动。软环境集中整治初见成效。市、县、乡三级政务公开不断深化，初步形成了常态长效工作机制。在市电视台进行了事业单位综合配套改革试点。

　　社会各项事业发展情况。全市共列入国家级星火、火炬计划4项，省级科技发展计划8项。加快了企业信息化步伐，为松江河林业局、市热力公司、方大集团等9户企业争取信息化专项贴息贷款5000多万元。我市被国际饮水资源保护组织命名为“中国·白山市长白山矿泉水资源可持续发展示范区”，人参被列入WTO原产地域产品保护范围，靖宇西洋参和抚松人参、临江黄芪中药材现代化基地分别通过国家和省验收。合理调整中小学布局，优化了教育资源配置，建成一批教育基础设施，开通了白山教育信息网，稳步推进了素质教育。加强了以农村文化为重点的文化建设。完成市区有线电视网络整合，新增16套电视节目。强化了有线电视网络安全防范，确保了安全播出。广泛开展全民健身活动，承办了国家和省级体育赛事3次，在十四届省运会上，金牌总数和团体总分均获第三名。医疗卫生事业健康发展，组建了白山中医集团。加强了人口与计划生育管理，稳定了低生育水平。制定了白山市妇女、儿童（2001—2010年）发展规划。

　　政治安定和社会稳定情况。严厉打击“法轮功”邪教组织非法活动，抓获一批顽固分子，捣毁10个地下窝点。建立了处置较大规模群体事件指挥协调系统，有效预防和妥善处置了各类群体性事件。严打整治斗争取得实效，刑事案件比去年下降8.3％，破案率提高3.2个百分点。以城区、农村、边境三项防范工程为重点，加强了社会治安综合治理，治安秩序进一步好转。侦破涉林刑事案件109起、治安案件1143起，严厉打击了毁林犯罪。狠抓了以煤炭行业为重点的安全生产工作。市长公开电话全年受理市民投诉1.87万件次，办结率达98％，畅通了政府与群众联系的渠道。加强了信访工作。认真处理了各类社会矛盾。

　　人民生活改善情况。预计全市城镇居民人均可支配收入、农民人均纯收入达到5540元和2917元，分别比去年增长8.9％和4％。千方百计筹措资金，为7662名下岗职工、6.4万离退休职工、22.2万城镇低保对象发放基本生活费、养老金和最低生活保障金共计5亿元。成功举办了首届房屋交易会，实现交易额近4000万元。加强了物业管理，筹集资金1000多万元，进行房屋维修和配套建设，改善了人居环境。市区供热质量得到提高。改善了城市公共交通基础设施。严厉查处制假售假违法行为，维护了消费者权益。

　　民主和法制建设情况。市政府自觉接受人大的法律监督、工作监督和政协的民主监督，制定了人大议案、建议和政协提案办理考核办法，全年共办理人大议案、建议和政协提案189件，办复率100％。依法强化了对经济责任、重点项目和企业的审计监督，规范了市场经济秩序，加强了宗教事务管理，维护了民族团结。

　　一年来，统计、环保、气象、人防、预备役、地震监测、外事、档案、市志、老龄、残疾人等工作都为全市经济社会发展做出了积极的贡献。

　　各位代表，市政府工作一年来所取得的成绩，是在市委的正确领导下，人大、政协、各民主党派、人民团体和无党派人士关心和支持的结果，是驻白山人民解放军指战员、武警部队官兵、公安司法干警、中省属企事业单位共同努力的结果，凝聚着全市各级干部和各族人民群众的心血和汗水。在此，我代表市政府表示衷心的感谢和崇高的敬意！

　　同时，我们也清醒地认识到，在发展过程中还存在一些深层次矛盾和问题，突出表现在：观念更新滞后，破解难题的能力不强；经济总量小，运行质量不高，发展后劲不足，全社会固定资产投资、财政收入和规模以上工业净利润没有完成预期目标，速度和效益形成较大反差；改革开放步伐不大，发展环境尤其是软环境亟待改善；就业和再就业压力越来越大，社会保障能力不强，部分低收入群众生活比较困难；有些部门的工作人员宗旨观念淡化，责任心不强，作风不实，服务质量差，工作效率低；个别领导干部为政不廉，以权谋私，贪污受贿，严重影响了政府形象；安全生产形势严峻，发生四起小煤矿重、特大事故，给人民群众生命财产造成了不可挽回的损失，作为全市安全生产第一责任人，我负有不可推卸的领导责任，在这里向全市人民做深刻检讨。

　　产生上述问题，既有客观方面的因素，更有主观上的原因。

　　从政府自身看，一是思想解放程度不够，缺乏开拓创新精神。虽然我们一再强调解放思想，但相当一部分干部小农意识、计划经济意识相当顽固，面对新形势、新任务，站位不高，思路狭窄，缺乏加快发展的责任感和紧迫感。二是虽然我们明确提出了突出工业主导地位的工作思路，但市政府领导班子，特别是我，在接待信访、尤其是大规模集体上访，处理棘手疑难问题，全力维护社会稳定方面，耗费了大量时间和精力，没能腾出更多的时间，集中更多的精力亲自抓新的项目，没有经常深入重点企业，帮助研究解决班子建设、市场开发、企业管理、企业信用等方面突出问题，导致工业经济运行质量不高、效益下降和缺少新的经济增长点。三是发展环境不宽松，成为制约经济发展的瓶颈。虽然市委、市政府下了很大决心整治软环境，但以经济建设为中心，合力兴工、合力兴市的思想意识远没有形成，在外地很容易办的事，在我们这里就是办不了，办不好。以权谋私、刁难勒索、吃拿卡要屡见不鲜，乱收费、乱摊派屡禁不止。个别部门和单位主动服务意识差，办事程序繁杂，工作效率低下。有利可图的事，一些部门越权执法，交叉执法，争着管，否则就推诿扯皮，敷衍塞责。对市委、市政府出台的政策措施，合意就执行，不合意就不执行。这种软环境严重制约了我市的经济发展。

　　从客观上讲，一是结构性矛盾突出。一方面，我市国民经济主要支撑和财政收入主要来源的工业，是以木材采运、林产品加工、矿产开发为主的资源输出型产业体系，受国家产业政策调整影响较大。实施天然林保护工程以来，木材采伐量由最高年份的300万立方米减少到今年的75万立方米。煤炭资源日渐枯竭，煤炭年产量由原来的600多万吨下降到400多万吨。再加上林矿产品的开发深度、科技含量、附加值低和向其它产业转移的速度慢，使结构性矛盾更加突出。在农业方面，大的龙头企业少，带动作用不强，产业化水平低。第三产业增加值占GDP的比重不高，而且层次较低，长白山旅游还没有形成产业规模。另一方面，占国民经济相当比重的国有企业体制改革迟缓，有些已经改制的企业机制转换不彻底，也没有发展起来。民营经济发展速度慢、比重低、大户少。整个国民经济的体制性障碍非常突出。二是受税收政策调整、消化虚数和其它因素的影响，导致财政短收，入不敷出。由于国家调整铁矿石资源税政策，改革企业所得税和个人所得税税制，加上天然林保护、清理整顿小煤矿等，使全市财政减收6500多万元。同时，迫于普涨工资、调职晋级、社会保障等刚性支出的压力，不得不寅吃卯粮。今年消化了财政收入虚数3300多万元，市本级财政已经没有虚数。

　　上述问题，已经引起我们的高度重视，市政府要在今后的工作中，认真加以解决。

　　二、全面建设小康社会的主要目标和2003年重点工作

　　本世纪头二十年，是我国全面建设小康社会的重要时期。根据党的十六大精神和省委、省政府的要求，市委四届二次全会确定了我市提前两年实现翻两番的全面建设小康社会目标：到2008年，国内生产总值比2000年翻一番，由85亿元增加到170亿元，年均增长9％；到2018年，国内生产总值再翻一番，达到340亿元，年均增长7.2％，人均国内生产总值超过3000美元。

　　——基本实现工业化，明显提高城镇化水平，缩小工农差距、城乡差别，全市综合经济实力显著增强

　　——社会就业比较充分，社会保障体系比较健全，居民家庭财产普遍增加，人民过上比较富裕的生活

　　——社会主义民主和法制更加健全，公民的政治、经济和文化权益得到切实保障，社会秩序良好，人民安居乐业。

　　——教科文卫事业有较大发展，全市人民的思想道德素质、科学文化素质和健康素质普遍明显提高，形成全民学习、终身学习的学习型社会，促进人的全面发展。

　　——可持续发展能力极大增强，环境保护水平和资源利用效率显著提高，走上生态效益、经济效益、社会效益和谐统一、文明发展的道路。

　　实现上述目标，任重而道远。我们一定要牢牢抓住这个可以大有作为的历史机遇期，在发展上有新思路，改革上有新突破，开放上有新局面，各项工作有新举措，经过全市人民坚持不懈地共同努力，实现全面建设小康社会目标。

　　2003年，市政府工作的总体要求是：以邓小平理论和 “三个代表”重要思想为指导，按照党的十六大精神和市委四届二次全会要求，围绕全面建设小康社会总体目标，紧紧把握发展这个第一要务，以结构调整为主线，以改革开放和科技进步为动力，以提高人民生活水平为根本目的，坚持“高效益、广就业、可持续”方针，进一步强化工业经济、城市建设和社会稳定三项重点工作，突出项目建设，增强发展后劲，提高经济运行质量，推进工业化、信息化、城镇化进程，加强社会主义政治文明和精神文明建设，实现国民经济持续健康发展和社会全面进步。主要预期目标是：国内生产总值增长9％，全社会固定资产投资增长8％，财政收入增长4％，城镇居民人均可支配收入和农民人均纯收入分别增长6％和4％，城镇登记失业率不超过4.4％，人口自然增长率控制在3.3‰以内。

　　完成上述任务，要重点抓好以下六个方面的工作。

　　（一）调整优化产业结构，提高经济运行质量

　　坚持在发展中调整结构，在调整中加快发展，扩充总量规模，提高运行质量。要把更多精力投入到项目建设上，抓好一批经济效益好，对区域经济拉动作用强的新项目。积极采用以信息技术为代表的高新技术、先进适用技术，加快新型工业化进程。继续提高能源、林产、矿产冶金、建材和塑料化工产业，培育医药、汽车配套产业，发展绿色食品、旅游产业，努力构筑具有我市特点的经济支撑体系。

　　加快结构调整步伐，实现工业经济效益恢复性增长。实现工业经济速度和结构、质量、效益的统一，要重点抓好六项工作：一是抓好重点企业的提速增效。明年，全市规模以上工业要实现增加值增长9％、净利润1.4亿元的目标，必须继续巩固铝厂、铝车轮、车门、金刚砂等企业良好的发展势头，力争满负荷生产，进一步提高经济效益。切实抓好天成、嵩华、喜丰等重点企业的增盈工作。下大气力抓好轴承重组后的启动，争取尽快恢复生产。着力挖掘困难企业中有潜力的车间或生产线，通过分块、分步启动，带动“双停”企业全面恢复生产。二是抓好建成项目的达产达效。组织专门力量对建成项目进行重点扶持，保证餐具纸、镁粉、微硅粉回收等技改项目全面启动生产，形成产能和效益。三是加快在建项目建设步伐。加大资金筹措力度，积极完善配套条件，力争节水器材、熊胆开明片、西洋参系列产品加工等项目年内投产。完成临江一次变电所建设，加快双河、聚宝、北江、双山六级等水电站和横山水利枢纽工程建设进度，力争用2—3年时间，形成地方水电网，提高转供和直供能力，牵动环保型冶炼企业快速发展。加快中药材现代化基地建设，积极推进医药企业GMP改造和认证，努力开发医药和保健新产品，尽快做大做强一批骨干企业。四是集中精力抓好新项目开发。建立后续项目储备库，加大国债和“双高一优”项目的争取力度，坚持政策性支持、银行信贷、市场融资相结合的筹资思路，促进后续项目的实施。成立重点项目攻坚组，力争用3—5年时间，新上几个支撑白山工业经济的好项目。五是全力支持国省属企业搞好生产经营，提高经济效益，发展接续产业，带动全市经济增长。借鉴和推广他们的管理经验，促进地方企业不断提高管理水平。六是积极推进信息化带动工业化进程，积极争取国家电子信息应用项目贴息贷款，建设白山市企业综合信息门户网站，实现互联互通，资源共享。

　　用工业化思维发展农业，推进农村经济工业化。我市农业和农村经济结构调整的重点是加快推进绿色食品、林地经济发展，不断提高工业化、产业化水平。以绿色食品城建设为牵导，扩大生产加工规模，在提高产品的质量和档次上下功夫，延长产业链条，积极培育国内、外市场，争创名牌产品。进一步抓好西洋参集团和永利药业保健品、康源公司绿色食品等龙头企业，发挥带动辐射作用；抓好农夫山泉、娃哈哈、泉阳泉、北光山野菜加工、济世保健品等重点项目的投产达效；积极创造条件，采取内引外联的形式，力争新上几户大规模绿色食品加工企业。按照“抓龙头、建基地、带农户、兴产业”的思路，全力推进林地经济向规模化、标准化迈进。大力推广林下栽参，尽快实现传统栽参模式向生态模式的转变。林地经济总收入占农村经济总收入的比重达到75％以上。增加优质高效作物种植面积，积极推行订单农业。加快牛、猪等肉类生产，积极开发鹿、野猪等特种养殖业，努力实现常规牧业向精品牧业的转变。

　　积极开发长白山旅游，牵动第三产业快速发展。旅游业要重点解决基础设施差、承载能力弱等突出问题，努力提高旅游产业的档次和水平。按照《白山市旅游发展总体规划》要求，加快编制县域旅游发展规划和重点景区开发规划。严格规划控制，规范景点建设，深入挖掘长白山四季景观，努力开发特色鲜明的旅游项目，办好长白山野生花卉旅游节、金秋枫叶节，积极开展冬季冰雪旅游，尽快形成四季旅游格局。进一步加快长白山大峡谷、望天鹅、龙山湖等景区道路、停车场、栈道等基础设施建设。以建设山水与建筑交融、人与自然和谐的长白山度假旅游基地为目标，高起点规划、高标准启动松江旅游度假区。坚持谁投资、谁受益的原则，鼓励和引导全社会兴办旅游业，实现投资主体多元化。不断完善吃、住、行、娱、购等旅游配套设施，努力提高服务质量。加强市场体系建设，拓宽流通渠道。继续推行仓储超市、物流配送、连锁经营等营销方式，有针对性地组织参加商交会、博览会和展洽会，大力发展社区服务等新兴服务业，促进第三产业的发展。

　　（二）加大基础设施投入，拉动国民经济增长

　　按照“积极而为，适度超前”的原则，采取向上争取一块、经营城市集中一块、合理举债筹措一块等办法，加大基础设施投入。一方面，扩大内需，刺激消费，提供就业岗位，增加财政收入，拉动近期经济增长，为工业经济发展赢得时间；另一方面，改善发展硬环境，增强载体功能，吸引生产要素集聚，推进城镇化进程。

　　加强城市建设与管理。遵循科学规划、重点建设、严格管理的原则，提高城市建设和管理水平。高起点、高质量地编制城市总体规划，拓宽城市发展空间。制定市区控制性、修建性详细规划。各县（市）区要尽快完善和编制县、乡、村三级规划。加强规划管理，严格审批权限，健全审批程序，确保规划的严肃性和权威性。积极探索把基础设施、公用服务设施作为资产来经营的新路子，通过出让收费权、收益权、冠名权等，拓宽融资渠道，实现城市建设投资多元化。把盘活土地资产作为经营城市的重点，按照规划要求，依法收储建设用地，通过公开招标、竞价出售，增强政府调控土地能力，搞活土地市场。采取积极的扶持政策，适当扩大房地产开发规模，带动建材、运输等相关产业发展。办好全市房屋展示交易会，活跃房地产市场。市区要着力抓好一区建设、一江改造和四个重点项目。“一区建设”即江北新区开发。要在调整新区规划的基础上，启动一期工程建设，筹措资金完善基础设施，逐步达到“六通一平”。“一江改造”即浑江市区段10公里改造工程。年内启动北岸堤路一体化、下游拦江蓄水钢板闸工程，用3—5年时间建成集交通、防洪、休闲、绿化和农业开发于一体的带状公园。四个重点项目是：开工建设白山大桥，带动新区开发；改造城市供水系统，提高供水能力；建设广播电视中心；建设消防指挥中心。进一步理顺城市管理体制，加强基础设施、环境卫生、公共秩序、社会服务等方面的管理，逐步形成常态管理机制。切实提高物业管理水平。按照强化中心城市、积极发展县城和中心镇、择优培育小城镇的思路，加快城镇化进程，重点加强县城、中心镇和旅游沿线小城镇的基础设施建设，增强辐射带动功能。

　　加快公路等基础设施建设。公路建设要按照突出中心、完善骨架、向外延伸、整体升级的总体要求，进一步优化城乡路网结构。一是突出市中心区路网建设。开工建设南线一级公路，为形成市区高等级环路奠定基础。二是继续完善路网骨架。抓好市县、县县通油路收尾工程，加快临江至长白二级公路、县城和重要集镇绕越公路建设进度，完成临江老岭隧道工程量的50％。三是全面启动向乡（镇）、旅游区和域外路网延伸工程。抓住国家支持县、乡公路建设的政策机遇，启动乡乡通油路工程，年内完成200公里建设任务，用3—4年时间使所有乡镇通油路。完成长白山西坡旅游公路，开工建设南坡旅游公路。加快靖宇至小西头公路建设，延伸至吉林；万良至沿江公路建设，延伸至延边；用几年时间，打通临江经三道沟至集安二级公路，延伸至丹东。开工建设市物流中心和靖宇铁路。加快浑江干流堤防配套改造工程和鸭绿江界河堤防建设。继续抓好全市农网改造二期工程。投资1.4亿元，完成白山移动通信宽带网、基站、直放站和白山通信网扩容工程。全面强化工程质量管理，严格实行建设项目监理制、招投标制和项目法人责任制，切实提高工程质量，坚决杜绝“豆腐渣”工程。

　　（三）推进改革开放，激发经济活力

　　继续深化改革，有效解决经济发展中的深层次矛盾。全面优化经济环境，加快招商引资步伐。积极调整所有制结构，释放发展能量，增强经济活力。

　　全面深化各项改革。大胆探索国有企业改革的新方法，进一步推进现代企业制度建设。加大国有企业破产核呆力度，稳妥实施已列入计划的市造纸厂、天时水泥刨花板厂的破产核呆工作，力争再有1—2户企业列入国家破产核呆计划。在嵩华纸业进行以国有净资产置换职工身份、转变国有企业性质试点，探索国有制的多种有效实现形式。采取措施，加快中小企业产权、股权出售和依法破产步伐。进一步完善股份制企业规范运作，实现经营的科学化和运作的规范化。认真总结集体企业资产量化、职工持股和经营者持大股的成功经验，积极稳妥地在具备条件的集体企业推广。在改革中，要坚持国有资产不流失、职工得到妥善安置、程序合法、有利于企业发展的原则，确保改革成功。继续深化农村税费改革，切实保证转移支付资金到位。在依法、自愿、有偿的基础上，加快土地流转进程，提高种植业规模化水平。稳步推进事业单位改革。继续深化城乡医疗卫生体制改革，抓好以大病救助为主的新型农村合作医疗制度试点工作。

　　推进政府自身改革，进一步优化发展环境。科学规范政府及部门的行政权力，加强对公共权力的制约和监督，提高管理的透明度，从根本上加大软环境整治力度。一是继续深化行政审批制度改革，清理、废止一批行政审批项目。提高政务服务中心运行质量，逐步由“一厅式”分散办理向“单窗口”联合办理转变。加快建设各县（市）区政务大厅。建成全市政府系统信息交换平台，实现市政府与各部门、与县（市）区政府之间的宽带连接，积极发展网上审批，推行电子政务，提高工作效率，降低行政成本。二是进一步规范会计核算制度改革，扩大核算范围，完善核算制度，科学制定支出限额和审批程序，从严控制不合理支出。三是加快土地收购储备制度改革，逐步形成土地收购、整理、储备、出让为一体的市场化机制。四是适时启动收费制度改革。通过以上改革，从源头上、机制上、利益关系上解决部门权力利益化、个人利益部门化等突出问题，全力营造有利于经济社会发展的服务环境、市场环境、信用环境、政策环境和法制环境。

　　进一步扩大对外开放，加快招商引资和民营经济发展步伐。立足白山资源和现有产业基础，以项目建设为重点，调整思路，拓宽渠道，加大招商引资力度。推出白山优势资源招商，推出国家已批准的“双高一优”项目招商，推出有发展前景但靠自身难以做大做强的骨干企业招商，推出发展潜力大又无能力开发的市场招商，提高招商引资的成功率。强化已签约项目的跟踪落实，切实提高合同履约率，尽快形成生产能力。启动北江经济开发区、靖宇生态经济开发区、松江河旅游经济开发区，组建开发区管委会，建立新型管理体制和运行机制，把工作重点放在营造宽松环境、提供优质服务上，吸引域内外资本向开发区集聚，成为新的经济增长点。临江、长白经济开发区要在现有基础上有新的进展。积极调整出口产品结构，努力实现外经贸和对朝边境贸易恢复性增长。把招商引资的重点放在民营资本上，既要着眼国际大企业、大财团，更要盯住国内知名和本市有投资能力的民营企业。鼓励民营企业参股、控股、购买、兼并国有或集体企业，实现低成本扩张，促进所有制结构调整。努力在全社会营造支持民营经济发展的良好氛围，真正使民营企业家政治上有地位，经济上得实惠，合法权益有保障，扶持和帮助民营经济做大做强。

　　（四）加强生态建设，走可持续发展之路

　　生态建设惠及长远，福荫子孙，是实现可持续发展的根本依托。我们要按照生态市建设总体规划，全面实施生态保护、恢复、治理工程，经过不懈努力，保护好我市的生态环境，在生态省建设中发挥龙头作用。

　　以加强保护区管理为重点，全面实施生态保护工程。长白山生态环境是我们赖以生存和发展的基础。要抓好长白山自然保护区、江源寒武—奥陶系界限地址自然保护区、长白冷水鱼自然保护区、长白山天然矿泉水靖宇水源保护区的规范和管理，对其它各县（市）区天然矿泉水等重要资源实行划区保护，严格管理。重点抓好森林资源、矿产资源和水资源的保护。继续巩固地方国有、集体林管护经营承包成果，采取国家利益与个人利益相结合的有效形式，积极探索和推进国有林的管护经营模式。全面加强林政管理，严厉打击滥砍盗伐等破坏森林资源和生态环境的不法行为。认真抓好森林防火工作，确保连续24年无重大森林火灾。按照“重保护、慎开发、可持续”的原则，严格实行矿产开发准入制，鼓励集约开发，规模经营，坚决打击非法开采经营活动。市政府要对矿泉水开发实行专管，重点支持大规模集约化生产，禁止10万吨以下小规模、低水平开发。

　　以植被恢复为重点，全面实施生态建设工程。快速推进20°以上坡耕地退耕还林和“三北防护林”四期工程建设，年内完成15万亩。积极争取国家林纸一体化项目资金，引导农民加快纸浆林建设速度。采取乔灌结合、针阔混交的模拟自然生态方式，启动城区环山绿化美化工程，力争用几年时间，完成市区、县城和重点乡镇周边荒山荒地的绿化美化任务。将市区划分为板石、河口、市郊、六道江四个区，集中力量分片建设。明年重点完成板石小区2818亩绿化任务。林业部门要做好任务分配、苗木选购和技术指导等准备工作，机关事业单位负责栽植，包栽、保活、保成林，一定5年不变。按照谁破坏、谁恢复的要求，组织专门力量对矿山、公路建设等破坏的植被进行踏查认定，逐地逐块落实治理恢复责任。新建项目必须把保护环境放在首位，及时恢复遭破坏的植被。今后要禁止公路、铁路两侧采石，严格规范河道挖沙。加强沟系开发管理，对已建和在建的生态沟，要强化管理和监督，真正做到保护而不破坏，支持生态型、复合型项目开发。不断加大小流域综合治理，减少水土流失，恢复生态功能。

　　以整治污染源为重点，全面实施污染治理工程。采取必要措施，坚决关停和治理对环境影响较大的小洗煤、小炼焦等高污染企业，对其它较大污染源，要限期治理，尽快达标排放。在全市范围内开展城市环境综合整治，解决好饭店、浴池、歌厅等严重影响居民生活的问题。市中心区内建筑工程一律使用商品混凝土，严禁现场搅拌。认真做好市污水处理厂和垃圾处理场的前期准备工作。

　　（五）发展各项社会事业，全面提高人的素质

　　加快发展，人是决定因素，要把全面提高人的思想道德、科学文化和健康素质，作为一项长期战略任务，努力促进人的全面发展。

　　全面提高人的思想道德素质。切实加强精神文明建设，认真贯彻公民道德建设实施纲要，继续强化社会公德、职业道德和家庭美德教育，坚持移风易俗，倡导科学、文明、健康的生活方式，全面开展科教、文体、法律、环保、卫生五进社区活动，深入持久地开展文明城市、文明村镇、文明行业、文明窗口创建活动，提高双拥模范城创建水平，努力提高广大群众的思想道德素质和城乡文明程度。围绕加快白山经济社会发展这一主题，在全体市民中广泛开展全面建设小康社会的宣传教育，凝聚人心，鼓舞斗志，为白山的经济发展和社会进步提供强大的精神动力。

　　全面提高人的科学文化素质。发展白山经济需要一大批掌握先进知识、具有较高科学文化素质的劳动者。教育作为发展科技和培养人才的基础，必须摆在优先发展的战略地位。要突出教育创新，深化教育改革，加快布局调整，优化资源配置，普及高中教育，加强职业技术教育，推进素质教育，逐步形成比较完善的国民教育体系。推广白山外国语学校公办民助的办学经验，进一步拓宽办学渠道。大力发展社会主义文化，不断丰富人们的精神世界。积极推进社区和农村文化建设，发展文化产业，广泛开展群众喜闻乐见的文化艺术活动，满足人民群众精神文化需求。进一步抓好广播电视工作，加强基础设施建设，努力扩大覆盖范围，利用省光缆把市广播电视节目传输到各县（市）。大力普及科学知识，弘扬科学精神，在全社会形成崇尚科学、鼓励创新、反对迷信和伪科学的良好氛围。

　　全面提高人的健康素质。切实加强医疗卫生基础设施建设，进一步提高各级医疗卫生机构的诊疗水平和服务质量，努力改善城乡居民的医疗、预防和保健条件。制定白山区域卫生规划，调整优化卫生资源配置，逐步建立健全覆盖城乡的医疗保健体系和卫生服务体系。继续抓好人口与计划生育工作，稳定低生育水平，提高人口素质。全面实施妇女、儿童发展规划。加强体育基础设施建设，广泛开展全民健身活动，努力提高竞技体育水平，力争在全国第十届冬运会上取得好成绩。

　　（六）切实维护群众利益，努力提高城乡居民生活水平

　　以扩大就业为中心，以帮扶弱势群体为重点，尽快建立起较为完善的社会保障体系，积极维护政治安定和社会稳定，努力营造安定祥和、安居乐业的社会环境。

　　积极拓宽就业渠道，提高社会保障能力。就业是民生之本。要加快发展社区服务业、劳动密集型产业和非公有制经济，积极启动“双停”企业，扩大劳务输出，努力创造更多的就业机会。完善就业培训和服务体系，提高劳动者就业技能。落实再就业有关优惠政策，重点扶持符合政策条件的国有企业下岗职工、享受最低生活保障的失业人员自谋职业和自主创业。引导富裕农民进城投资兴业，支持城镇富余劳动力下乡，发展以种、养、加为主的农村经济，促进城乡劳动力双向流动。认真落实“两个确保”政策，多渠道筹措资金，确保在中心的下岗职工基本生活费、离退休人员养老金按时足额发放；进一步扩大养老保险覆盖范围，健全失业保险制度，加大基金征缴力度，逐步使国企下岗职工基本生活保障向失业保险并轨。增加社会保障资金投入，积极争取国家转移支付资金，努力使城镇贫困人口的基本生活得到最低保障。做好农村扶贫工作。扩大基本医疗保险覆盖面，提高基本医疗保障水平。立足于保工资、保稳定、保重点支出，优化财政支出结构。金融部门要在防范金融风险的基础上，加大对地方经济发展的支持力度。

　　全力维护政治安定和社会稳定。坚持“打防并举，预防为主”的方针，重点抓好城市中心区，扼制案件多发区，稳定边境地区，全面加强社会治安综合治理，加快防控体系建设，建立常态长效运行机制。加强信息情报工作，完善处置预案，提高对突发事件的处置能力。继续深入开展严打整治斗争，深挖黑恶势力，严厉打击暴力犯罪、毁林犯罪、跨国犯罪、经济犯罪和危害人民群众生命财产的盗窃、抢劫、诈骗等多发犯罪。加强国家安全工作，警惕境外敌对势力、敌对分子的渗透和破坏活动。加强广播电视网络监管，确保运行安全。防范和惩治“法轮功”等邪教组织的犯罪活动。依法加强宗教事务管理。进一步维护民族团结。

　　下大气力抓好安全生产。安全生产责任重于泰山。各级政府必须增强责任感和紧迫感，高度重视安全生产工作，严格管理，狠抓落实，彻底扭转安全生产被动局面。全面贯彻安全生产法，进一步强化全市安全生产监管体系建设。重点整治煤炭行业的安全隐患，加强对整改合格井的安全监管，坚决关闭不合格矿井，严厉打击非法盗采，彻底摧毁非法矿井，依法从重从快惩治非法矿主，坚决遏制住事故频发势头。进一步加强非煤矿山、危险化学品、建筑施工、交通运输、校园、网络安全和公众聚集场所消防等专项整治，严防重大事故发生。认真落实安全生产责任追究制，严格依法追究事故责任者。把取缔非法“黑矿”和安全生产责任追究与打黑除恶、惩治腐败紧密结合起来，依法严惩“靠山”和“保护伞”。

　　努力做好信访工作。从讲政治、保稳定的大局出发，以对人民群众高度负责的精神，认真接待群众来信来访。严格落实信访工作承诺制、任务交办制、法律服务制、责任追究制和领导信访接待日制度。按照分级负责、归口办理的原则，进一步明确信访工作责任，提高处理疑难问题的能力，坚决杜绝推诿扯皮现象。切实做好群体性信访事件的超前预测、超前排查、超前疏导，把矛盾和问题解决在基层，化解在萌芽状态。

　　三、以“三个代表”重要思想为指导，全面加强政府自身建设

　　全体公务员特别是各级领导干部，要深入学习贯彻十六大精神，认真践行“三个代表”重要思想，深怀爱民之心，恪守为民之责，善谋富民之策，多办利民之事，努力建设勤政、廉洁、务实、高效的政府机关。

　　解放思想，与时俱进。全面建设小康社会，加快经济和社会事业发展，思想解放必须先行。各级政府和政府各部门领导干部，必须顺应加快发展的历史潮流，把思想解放定位到时代发展的前沿，用新视野认识新事物，用新观念研究新问题，用新思路破解深层次矛盾，切实打破因循守旧的思维定势，彻底摒弃小农意识、计划经济意识和官本位思想，不断提高驾驭市场经济的能力和水平。解放思想，关键在于打破常规，勇于创新。不创新就不能突破，不创新就难以发展。要从根本上解决怕担风险不敢创新、维护既得利益不想创新、水平低不会创新等突出问题。按照“三个有利于”的标准，积极探索，大胆实践，放手让一切劳动、知识、技术、管理和资本的活力竞相迸发，让一切创造社会财富的源泉充分涌流，造福白山人民。

　　勤政务实，执政为民。发展是硬道理，一切矛盾和问题都要靠不断发展去解决。怨天尤人无济于事，不真抓实干没有出路。各级政府必须把加快发展作为执政兴市的第一要务，牢固树立强烈的进取意识，增强时不我待、不进则退的紧迫感。各级领导干部都要树立为官一任、造福一方的责任感，在其位，谋其政，尽其责，努力做到科学决策、少留遗憾，多攒家底、少留债务，多解难题、少留包袱。大力提倡少休息、多工作，少花钱、多办事，少享受、多奉献，集中精力解决改革发展稳定中的主要矛盾，解决关系人民群众切身利益的实际问题。适应加入WTO要求，逐步把政府职能转到经济调节、市场监管、社会管理和公共服务上来。进一步转变思想和工作作风，坚持实事求是，深入基层，深入群众，说实话，报实数，办实事，求实效，把各项工作落到实处。

　　依法行政，从严治政。大力推进政治文明建设，自觉接受人大的法律监督、工作监督和政协的民主监督，认真听取民主党派、工商联、无党派民主人士和人民团体的意见，支持工会、共青团、妇联等群团组织按照各自的章程开展工作。进一步强化市、县、乡三级政务公开，推进政府职能转变，使行政权力运行真正置于法律监督和社会监督之下。按照政府采购法要求，理顺政府采购体制，加强监督管理。积极推进村民和社区居民自治。各级政府要从严治政，严肃政治纪律、组织纪律和工作纪律，树正气，压歪风，带好班子，管好队伍，做到令行禁止，确保政令畅通。

　　克已奉公，廉洁自重。要认真吸取我市个别领导干部违法犯罪的深刻教训，正确对待自己手中的权力，讲党性、讲官德、讲人格，增强自觉抵制各种腐朽思想侵蚀的能力，切实做到以身作则，自省自重，警钟长鸣。认真落实党风廉政建设责任制，突出抓好领导干部廉洁自律，查处大要案，纠正部门和行业不正之风。进一步规范政府的行政权力和行为，强化领导干部任期经济责任审计，通过体制和制度创新，以反腐败的实际成果，取信于民，把党风廉政建设提高到一个新水平。

　　各位代表，党的十六大为我们描绘了全面建设小康社会的宏伟蓝图，市委四届二次全会确定了我市今后一个时期的发展目标。我们要聚精会神搞建设，一心一意谋发展，正视困难不气馁，面对艰险不后退，凝聚起全市132万人民的智慧和力量，同心同德，卧薪尝胆，开拓创新，发愤图强，用辛勤的劳动和汗水，创造我们更加美好的明天！
